# Sounds can boost the awareness of visual events through attention without cross-modal integration

**DOI:** 10.1038/srep41684

**Published:** 2017-01-31

**Authors:** Márta Szabina Pápai, Salvador Soto-Faraco

**Affiliations:** 1 Multisensory Research Group, Center for Brain and Cognition, Universitat Pompeu Fabra, Barcelona, Spain; 2 Institució Catalana de Recerca i Estudis Avançats (ICREA), Barcelona, Spain

## Abstract

Cross-modal interactions can lead to enhancement of visual perception, even for visual events below awareness. However, the underlying mechanism is still unclear. Can purely bottom-up cross-modal integration break through the threshold of awareness? We used a binocular rivalry paradigm to measure perceptual switches after brief flashes or sounds which, sometimes, co-occurred. When flashes at the suppressed eye coincided with sounds, perceptual switches occurred the earliest. Yet, contrary to the hypothesis of cross-modal integration, this facilitation never surpassed the assumption of probability summation of independent sensory signals. A follow-up experiment replicated the same pattern of results using silent gaps embedded in continuous noise, instead of sounds. This manipulation should weaken putative sound-flash integration, although keep them salient as bottom-up attention cues. Additional results showed that spatial congruency between flashes and sounds did not determine the effectiveness of cross-modal facilitation, which was again not better than probability summation. Thus, the present findings fail to fully support the hypothesis of bottom-up cross-modal integration, above and beyond the independent contribution of two transient signals, as an account for cross-modal enhancement of visual events below level of awareness.

How many of us have stopped in our tracks at the sudden car honk while absentmindedly crossing a street? Such dramatic attention capture illustrates the action of the exogenous attention system, quickly summoning mental resources when events potentially require imperative action. Attention-grabbing sensory events can express in several sensory modalities^1^ and operate even when completely task-irrelevant^2^. Remarkably, it has been suggested that signals from different sensory modalities might integrate pre-attentively, and result in stronger, automatic capture^3^. For example, a spatially uninformative auditory stimulus can improve the selection of a concurrent visual target amongst visual distracters^4^,^5^; though see^6^. What is often at stake in these claims of automaticity is the assertion that multisensory integration can proceed in its full strength in the absence of endogenous, top-down modulation of attention.

Here, we address a strong test of automaticity for multisensory integration: Whether an unconscious visual event can be integrated with a concurrent sound. Some forms of cross-modal interaction have been already reported to occur below awareness in patients^3^,^7^,^8^,^9^ and in healthy observers under conditions of different suppression depth, including masking^10^, priming^11^ (though see^12^), repetition blindness^13^ and, attentional blink^14^. A convenient approach to understand the limits of visual awareness is binocular rivalry (BR), a phenomenon that has been long of fascination to researchers because it dissociates sensory stimulation from perceptual experience^15^. BR emerges when each eye receives incompatible images, and it is subjectively experienced as stochastic dominance fluctuations between the two images over time. Thus, binocular rivalry can be used to investigate processing of stimuli that are physically present but remain unaware, that is, suppressed^15^.

A variety of studies have addressed whether cross-modal events can indeed influence the dynamics of BR^16^,^17^,^18^,^19^,^20^, including those using continuous flash suppression^17^,^21^,^22^. One crucial aspect here, in order to answer the initial question about automaticity of multisensory integration, is to rule out the influence of top-down endogenous attention effects, also known to modulate BR. These top-down mechanisms can potentially explain why a non-visual stimulus exerts an influence on the dominance of one or another visual percept in BR. Despite it is clear that switches of perceptual state can be triggered by transient stimuli^23^,^24^, the significant impact of endogenous attention in BR is also well-known. For example, an observer can voluntarily modulate relative dominance times between rival percepts^25^,^26^ or change alternation rate^27^ and, what is more, alternation can even stop altogether in the absence of top-down attention^28^,^29^.

Many studies using BR to address cross-modal influences on unconscious visual stimuli may reflect either kind of effect, or a combination of the two. For instance, when an auditory stimulus is (spatially, temporally, semantically…) contingent on one of the two rival visual stimuli^26^,^30^,^31^, the dynamics of BR might be altered via well-known top-down influences such as prediction and expectation^26^. In these cases, a particular time of appearance, a particular visual feature, or a particular location is anticipated and hence receives top-down priority in the suppressed percept, producing a quicker emergence to consciousness. These kinds of influence are even more likely in cases when the presentation of the two corresponding stimuli (non-visual and visual) is sustained throughout a period of time, given the extended opportunity for such top-down mechanisms^31^ to operate. These kinds of top-down mediated processes, though interesting in their own right, do not allow one to give a conclusive answer to the initial question of automaticity in the sense of whether bottom-up multisensory integration, for unconscious visual stimuli, is pre-attentive.

Indeed, a few studies have controlled for the role of voluntary strategies based on the correspondence between stimuli in the two modalities, hence avoiding a major role of expectation^17^,^20^,^32^. Zhou *et al*.^17^ explicitly tested the role of expectation for cross-modal correspondence (i.e., participants sniff a neutral odour, but told they are smelling an odour corresponding to the suppressed visual stimulus), and did not find a multisensory effect in that case. Lunghi & Alais^32^ demonstrated a tactile influence on suppressed visual gratings with such fine selective orientation tuning that participants were unable to resolve it when asked explicitly in a subsequent, suprathreshold, visual task. Additionally, Lunghi *et al*.^20^ used shallow modulation depth where, regardless of expectation, cross-modal stimuli were too weak to bias BR by their own. One particularly striking finding in these studies is the exquisite selectivity of the effects, constrained by very precise cross-modal feature matching (such as spatial orientation or modulation depth). This sharp feature tuning of cross-modal effects suggests that these cases of cross-modal integration of suppressed visual stimuli may not be based on the kinds of mechanisms responsible for quick, bottom-up capture of attention, which are typically driven by low-level temporal and/or spatial coincidence, but less specific about correspondence between higher-level stimulus features. Interestingly, based on the Integrated Information Theory of Consciousness^33^,^34^, these congruency effects may actually reflect the role of consciousness in integrating new associations. According to this theory, existing links between stimuli can operate unconsciously only when these associations have been developed and strengthened a priori through multiple, conscious exposures. By contrast, new links between stimuli cannot be established below level of awareness. One might question then, what if cross-modal stimuli were not associated in any high-level features, other than temporal coincidence?

Thus, in the present study we try to understand if cross-modal integration occurs below level of awareness in an immediate bottom-up fashion so that the integrated percept summons attention orienting independently of endogenous processes (see^35^ for similar effects in full awareness). This can be tested using abrupt stimuli (i.e., appearance or cessation thereof) where cross-modal coincidence is neither task-relevant nor predictive in time, space or feature content of the rival stimuli presentation^5^, and whose correspondence is limited to temporal coincidence. Actually, temporal coincidence has been put forward as the ultimate feature to support automatic, attention-free multisensory interaction that is independent on prior context, objectness of the events, or goal of the observer (including task relevance)^36^.

We measured the alternation dynamics in a BR paradigm as a function of abrupt visual events (presented in the suppressed or dominant eye) and spatially uninformative sound events, presented alone or synchronized. Above and beyond temporal coincidence at onset, bimodal stimuli in the present experiments do not share any feature-based congruency, nor are predictive of each other. Under such conditions, we tried to find out whether bottom-up cross-modal integration mechanisms can operate on suppressed visual information, hence ‘rescuing’ unconscious visual events into awareness. According to an automatic integration hypothesis, when a suppressed visual event coincides with an abrupt sound, it should trigger a switch in dominance toward the suppressed eye with more strength than visual or audio events alone. In order to conclusively infer multisensory integration, eventual cross-modal facilitation should surpass the combined probability of each sensory signal contributing independently to a switch in BR dominance.

## Results

**Figure 1:**
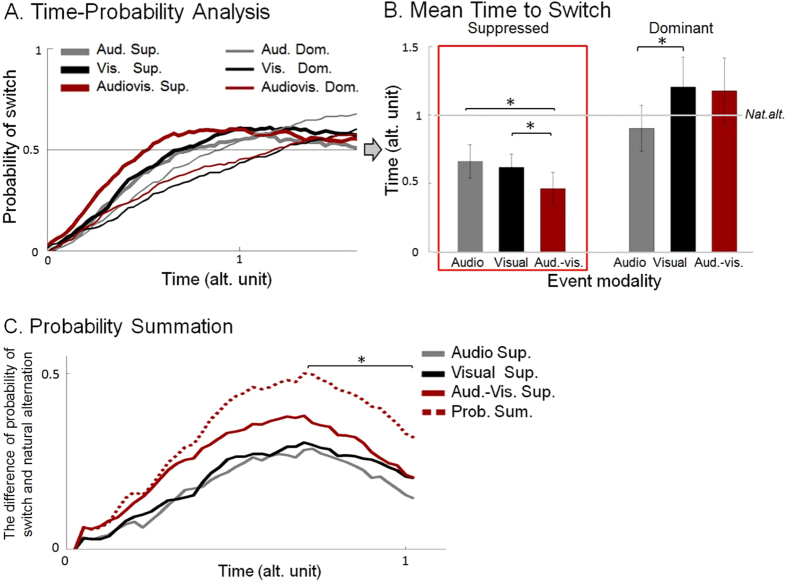
(**A**) *Time-Probability Analysis.* The probability of switch is plotted as a function of relative time, expressed in alternation units (sampling points of 0.025 alt. unit). Switch in suppressed condition refers to change in perceptual state from seeing the radial checkerboard to seeing the Gabor (as events were presented on Gabor patch under suppression; Audio suppressed, Visual suppressed, Audiovisual suppressed). On the other hand, switch in dominant condition indicates change in perceptual state from seeing the Gabor to seeing the radial checkerboard (as events were presented on Gabor patch under dominance; Audio dominant, Visual dominant, Audiovisual dominant). Zero time point indicates event onset time, which never occurred during piecemeal percept. (**B**) *Mean Time to Switch (time at which probability crosses 50%*). The bars indicate the mean relative time of switch (in alternation unit with SEM) in each condition separately. (**C**) *Probability Summation*. The curves indicate differences between different suppressed conditions (Audio suppressed, Visual suppressed, Audiovisual suppressed, respectively) and the baseline alternation (probability of switch when Gabor is suppressed in natural alternation runs), as well as the curve estimated from the PSM (Prob. Sum., see Probability Summation). Additionally, please note that significant comparisons marked by ‘*’.

Participants were exposed to a BR situation between a Gabor patch and a checkerboard display. Dominance was individually adjusted so that the checkerboard would be dominant ~70% of the time, hence resulting in deeper suppression of the Gabor. At pseudo-randomly chosen moments (see Methods) one out of several events could occur: a flash at the Gabor when it was dominant (VD) or when it was suppressed (VS), a sound while the Gabor was dominant (AD) or while it was suppressed (AS), or a combination of sound and flash, again under Gabor dominance or suppression (AVS, AVD, respectively). The focus of interest is on the conditions where the Gabor is suppressed, and the measure consists of changes in perceptual state following events presented on the suppressed Gabor patch, while participants report seeing the radial checkerboard (AVS: audiovisual suppressed, VS: visual suppressed, AS: audio is presented as Gabor patch is suppressed). Dominant conditions were introduced to balance out stimuli (in order to avoid potential expectation regarding event onsets on suppressed conditions), and refer to when events are presented on the currently seen Gabor patch (AVD: audiovisual dominant, VD: visual dominant, AD: Gabor patch was dominant at the moment of audio presentation) (Fig. 1A). The Mean Time to Switch (MTS) index (see Fig. 1B) helps compare latency changes in switch probability after each particular event of interest. Short MTS latencies reflect quick emergence of the suppressed percept into awareness. Long MTS latencies reflect deeper suppression and a prolonged perceptual state of the current dominant percept. In particular, the contrasts of interest are whether, under Gabor suppression, the bimodal event produces shorter MTS than either of the unisensory event; that is, the hypothesis under test can be expressed: ((MTS_AVS_ < MTS_AS_) AND (MTS_AVS_ < MTS_AV_)). To test this hypothesis, we will use a one-tail t-test (α = 0.05) for each term of the contrast, with the criterion for significance of each contrast corrected for the number of contrasts (two), according to Bonferroni (α/2 = 0.025).

### Experiment 1

We ran a Repeated Measures ANOVA (with Greenhouse-Geisser correction where appropriate) on the Mean Time to Switch (see Methods) latency data with the following, within participants’ factors: percept dominance (*Gabor dominant, Gabor suppressed*) and event modality (*audio, visual, audiovisual*). The ANOVA returned a main effect of percept dominance F(1,11) = 13.088, p = 0.004, whereby events presented while the Gabor is suppressed tend to produce a quicker switch (e.g., make the Gabor percept emerge to awareness) than when the Gabor is already dominant (e.g., make the Gabor stay dominant). Yet, important for the purposes of the study, a significant interaction F(1.292,14.208) = 7.705, p = 0.011 granted further analysis. Planned paired t-tests were used as to test the main hypothesis concerning the effect of events under Gabor suppression, as described above. Supporting the presence of cross-modal effects, audiovisual events presented under suppression induced an earlier emergence of the suppressed percept to awareness, compared to visual alone and audio alone events t(11) = −2.925, p = 0.007 and t(11) = −2.38, p = 0.0185, both tests one-tailed, significant, after Bonferroni-corrected α/2 = 0.025. The comparison between visual and audio only MTS was not part the tested hypothesis, and its outcome is neutral to the main rationale of the hypothesis. Yet, for completeness, we ran a two-tailed paired t-test and found no difference between them, p = 0.386.

For completeness we looked at mean switch latencies when events were presented under dominance (i.e., when the Gabor was dominant). Visual events presented under dominance led to later switch times (i.e., prolonged the dominance) compared to audio alone events, t(11) = 4.588, p = 0.001. There were no significant differences between visual and audiovisual conditions p = 0.577, either between audio and audiovisual conditions p = 0.049 though. These t-tests were two-tailed (α = 0.05; given that there was no a priori hypothesis about the direction of the difference), and significance criterion corrected after Bonferroni α/3 = 0.016.

Furthermore, visual events presented on the suppressed percept resulted in faster switches than when it is presented under dominance t(11) = −4.408, p < 0.001. The audiovisual events acted in the same way, indicating faster switches when presented under suppression t(11) = −3.806, p < 0.001. Between audio conditions there was no statistically significant difference, p = 0.202. Please note that, contrary to visual events presented at one eye, the auditory event did not go through a monocular channel, but only coincide in time with one or another perceptual state (Gabor suppressed or Gabor dominant). Hence its effects were equivalent in both perceptual states. These tests were two-tailed, without having hypothesis about their direction, Bonferroni-corrected α/3 = 0.016.

The pattern of switch times for the suppressed conditions is consistent with the hypothesis of automatic multisensory integration. That is, the effect of an AV event on switch times from suppression seems to be stronger than the effects of each of the two events separately. In this case the implication of top-down mechanism is unlikely because of the lack of temporal, feature-based and spatial contingency. However, before accepting the conclusion of unconscious cross-modal integration, one must consider the possibility that the multisensory advantage is a consequence of the independent contributions of each unisensory event in the audiovisual trials, rather from a unified multisensory event. This alternative interpretation has been considered in other multisensory enhancement phenomena, and it has been formalized with the probability summation benchmark^37^,^38^,^39^,^40^. We modelled the Probability Summation Model (PSM) time course based on the data from unisensory events during Gabor suppression, together with the individual natural alternation rate (see Methods). The statistical comparison between the PSM and the empirical AV suppressed data over time (Fig. 1C) did not suggest a violation of the model. If something, some significant differences between the empirical and theoretical time-courses, by the end of the time window (0.725 to 1.025 time unit, p < 0.05), indicated that the probability of a switch after audiovisual events dropped below the threshold set by the model.

Considering the Integrated Information Theory of Consciousness^33^,^34^ one might assume that associations can operate even below level of awareness only after they have been consciously established. Thus, in order to test whether any multisensory integration effects might have been built over time in our protocol, based on the events presented under dominance, we ran new tests against the PSM, separately for data from the first and second half of the experiment. None of the tests surpassed the limit of statistical facilitation^38^ as indexed by the PSM (see Supplemental Material, *Probability Summation tests by experiment half*), indicating no effect of training (at least, when it is implicit and over the course of the five one hour experimental sessions of the study).

Thus, according to Experiment 1, a flash on the suppressed eye induced a faster switch when coinciding with a sound than the flash or the sound per se, while unisensory events did not differ from each other. However, the speed up in rivalry switches induced by bimodal stimuli was well within what would be expected assuming independent contributions of vision and sound (from a PSM). Hence, the integration account cannot be concluded. Additionally, please note that our protocol affords comparison with prior findings in the BR literature, in terms of some known effects. First, for visual only events, we observed that a flash on the suppressed percept speeded perceptual switch compared to a flash presented on the dominant percept^25^. Second, the effect of sound events was similar to that of a binocularly presented flash presented in the background^23^, that is, it produced general alerting effects speeding up switches regardless of which visual percept was currently seen (Supplemental Material, *MTS (Mean Time to Switch) in experimental conditions vs. natural alternation)*.

### Experiment 2

After seeing that the cross-modal advantage did not satisfy the PSM, one might assume that the effects seen in Experiment 1 can be attributed to exogenous attention mechanisms being summoned by each of the abrupt unisensory stimuli independently. Nevertheless, the threshold provided by probability summation is just a benchmark, instead of a test for the lack of integration. Technically, multisensory integration still might have occurred, albeit expressed below probability summation. Thus, we conducted a new version of the study with a new scenario designed to minimize multisensory integration and capitalize on bottom-up attentional mechanisms, in order to disentangle attentional cueing from multisensory integration as much as possible. To do so, we replaced the sounds used in Experiment 1 with a transient gap of silence, embedded in continuous white noise. In this way, the audio event stayed robust in terms of saliency, and remained a potential candidate for bottom-up attentional capture. Instead, in terms of potential for integration with visual events, sound absence is weaker than sound presence. Precisely because these poorer conditions for integration, if there is some multisensory integration underlying the cross-modal effect seen in Experiment 1, then it should be reduced in Experiment 2. If the outcome of Experiment 2 is like in Experiment 1, then the account based on the effect of individual contributions of the stimuli by bottom-up attentional capture will be confirmed.

**Figure 2:**
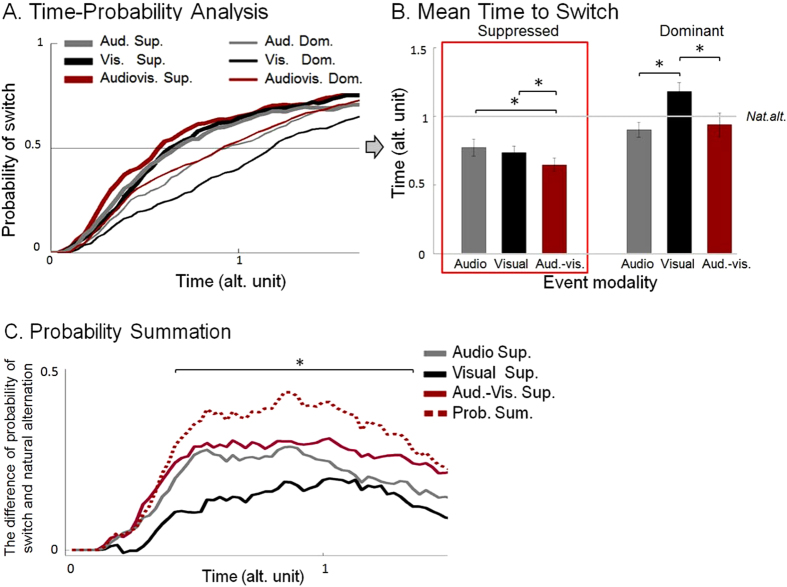
(**A**) *Time-Probability Analysis.* The probability of switch is plotted as a function of relative time, expressed in alternation units (sampling points of 0.025 alt. unit). Events were presented on Gabor patch under suppression (Audio suppressed, Visual suppressed, Audiovisual suppressed) and, on Gabor patch under dominance (Audio dominant, Visual dominant, Audiovisual dominant). Please note that audio event is a gap in noise. Zero time point indicates event onset time, which never occurred during piecemeal percept. (**B**) *Mean Time to Switch (time at which probability crosses 50%*). The bars indicate the mean relative time of switch (in alternation unit with SEM) after in each condition separately. (**C**) *Probability Summation*. The curves indicate differences between different suppressed conditions (Audio suppressed, Visual Suppressed, Audiovisual suppressed, respectively) and the baseline alternation (probability of switch when Gabor is suppressed in natural alternation runs), as well as the curve estimated from the PSM (Prob. Sum., see Probability Summation). Additionally, please note that significant comparisons marked by ‘*’.

A Repeated Measures ANOVA run on Mean Time to Switch (MTS) latency data of Experiment 2 (Fig. 2) revealed a pattern very similar to Experiment 1. The main effect of perceptual state *(Gabor dominant, suppressed)* F(1,11) = 17.097, p = 0.002 showed that events on the suppressed percept speeded up perceptual switch (pulled the percept into awareness), compared to events presented on the dominant percept (which, prolonged dominance). There was a main effect of event modality (*audio, visual, audiovisual*) F(2,22) = 7.038, p = 0.004, indicating that audiovisual events led to quicker switches than visual events t(23) = −4.504, p < 0.001, however audiovisual events in general did not produce faster switches than sounds t(23) = −1.254, p = 0.222, two-tailed paired t-tests, Bonferroni-corrected α/2 = 0.025. Critically, there was also significant interaction between F(2,22) = 6.895, p = 0.005, which granted further analysis. We then ran the planned contrast for our main hypothesis, on the MTS for events occurring with the suppressed Gabor. Perceptual switch happened earlier (MTS was shorter) following audiovisual events than visual alone t(11) = −3.256, p = 0.004 and audio alone events t(11) = −2.484, p = 0.015, both significant after Bonferroni correction of the two one-tail t-tests, α/2 = 0.025. For completeness, we ran an additional two-tailed t-test between visual only and audio only MTS, which turned out to be not different from each other, p = 0.41, suggesting that their strength was similar (Fig. 2A,B). This replicates the pattern found in Experiment 1.

Like in Experiment 1, we run further exploratory tests for MTS to events presented under Gabor dominance, and the two-tailed t-tests with Bonferroni-corrected α/3 = 0.016 suggested that the mean switch latency was significantly longer after visual only events, compared to audiovisual t(11) = −3.681, p = 0.004, and audio only events t(11) = −4.359, p = 0.001. Switch latency in audiovisual conditions were no different from the ones in audio only condition, p = 0.905 (Fig. 2A,B).

Regarding the reality check tests, perceptual switch happened sooner in suppressed conditions compared to dominant in visual and audiovisual conditions, respectively t(11) = −5.181, p < 0.001 and t(11) = −3.571, p = 0.004. However, audio events were not different under dominance or suppression t(11) = −2.473, p = 0.031. These t-tests were two-tailed (α = 0.05; given that there was no a priori about the direction of the difference), and significance criterion corrected after Bonferroni α/3 = 0.016.

Critically, audiovisual empirical data never surpassed the threshold set by the PSM (in fact, empirical AV data were significantly below the model from 0.375 to 1.325 alternation units, p < 0.05) (Fig. 2C). Thus, the analysis of the data showed that the faster alternation induced after cross-modal events when the stimulus is below awareness does not go beyond what is predicted by the PSM. Consequently, we cannot discard that the AV facilitation in switch latency occurred because of the independent contributions of unimodal stimuli.

### Analysis of Exp 1 and Exp 2

By hypothesis, we reasoned that if there is some certain multisensory integration underlying the cross-modal effect of Experiment 1, albeit expressing below the probability summation threshold, then this effect should be eliminated or reduced in Experiment 2, due to the poorer conditions for multisensory integration. In order to compare the data from the two experiments we ran a Repeated Measures ANOVA on the MTS including the within participants factors defined as before, percept dominance (*Gabor dominant, suppressed*) and event modality (*audio, visual, audiovisual*) and the between participants factor experiment (*Exp1, Exp2*). The main effect of experiment did not reveal significant effects F(1,22) = 0.007, p = 0.935. Moreover, there was no interaction between experiment and percept dominance F(1,22) = 1.712, p = 0.204, neither with event modality F(2,44) = 0.535, p = 0.59. Because the interaction between the three factors (i.e. *experiment, percept dominance and event modality*) F(1.355, 29.805) = 3.297, p = 0.068, was near significant, we decided to follow it up by analysing the dataset for suppressed and dominant percepts separately, with the factors modality and experiment in each. The partial analysis focusing specifically on the suppressed percept data returned a significant main effect of modality F(2,44) = 6.914, p = 0.002, explained by the pattern in the previous analyses, and no main effect F(1,22) = 1.927, p = 0.179 nor interaction with experiment F < 1. The origin of the near-significant three-way interaction from the overall analysis was therefore, the dominant data, in which the significant main effect of modality F(2,44) = 10.690), p = 0.001 was modulated by a near-significant interaction with experiment F(2,44) = 8.169), p = 0.07. This pattern in the dominant data reflected that, in Experiment 1 (sound beep), the AV events tended to pattern like the visual only condition (long MTS), whereas in Experiment 2 (sound gap) AV events tended to pattern like the auditory events (short MTS). But, for the sake of data interpretation, the main result to arise from this pooled analysis is that there is no difference in the magnitude of the cross-modal facilitation between the two experiments, for the visual suppressed percepts.

In a further attempt to evaluate inter-experiment differences, we re-calculated the individual audiovisual suppressed and audio suppressed MTS latencies with respect to the visual suppressed MTS, in both experiments. This provides a relative score for the conditions of interest, calculated with respect to a physically identical condition in both experiments (*visual*), thus helping to remove inter-experiment and inter-participant variability and focus on possible differential effects of sound and the sound-vision interaction. In this case, the factor event modality had only two levels (*audiovisual and audio*). This ANOVA reproduced the same pattern as before: A main effect of modality F(1,22) = 10.251, p = 0.004 whereby audiovisual events produced significantly shorter MTS latencies than audio alone events, but there was a lack of significant main effect of experiment F < 1 or interaction F(1,22) = 1.253, p = 0.257.

Given that in Experiment 2 the chance for multisensory integration was putatively reduced by the use of gap stimuli, but the advantage of multisensory events remained, the data support again an attentional capture account over and above a multisensory integration one. In support for this attentional explanation, which assumes independent contributions of two unisensory signals, the speed up effect granted by multisensory events was below the threshold predicted by PSM. Thus, data from Experiment 2 converges on the conclusion of independent contributions of bottom-up saliency signals from transient events. An important consideration at this point is that the interpretation above is based on the assumption that absence of stimulation (i.e., silent gap) will reduce the chances for (or prevent) multisensory integration. Despite this kind of logic has been used in other studies^41^, the assumption may be controversial, and will be addressed in more detail in the Discussion.

### Experiment 3

One could raise the question that multisensory integration is often maximal (if not limited to) when the component stimuli occur close in space, as well as in time^7^,^42^. In our two first experiments, the location of the sound was centred horizontally, with the loudspeaker located below the screen, whereas the visual event (flash) happened centred horizontally but was presented in the upper and lower halves of the monitor, hence close but not matched to the location of the sound (i.e. spatial discrepancy of 30.3° and 28.3°, respectively). Based on the spatial rule of audiovisual integration^43^, this discrepancy might offer a possible alternative explanation of the lack of evidence for integration in Experiments 1 and 2. This alternative account of our data is somehow weakened by positive evidence of effective cross-modal cueing^1^ even with slight spatial discrepancies (14° in elevation). Furthermore, as some studies claim, the relevance of the spatial rule might be constrained by task-related factors^44^. Still, in order to provide an empirical answer to this concern we reproduced our experiment but using congruently vs. incongruently located auditory events (this time noise bursts), in a new Experiment 3, adapted from Experiment 1.

**Figure 3:**
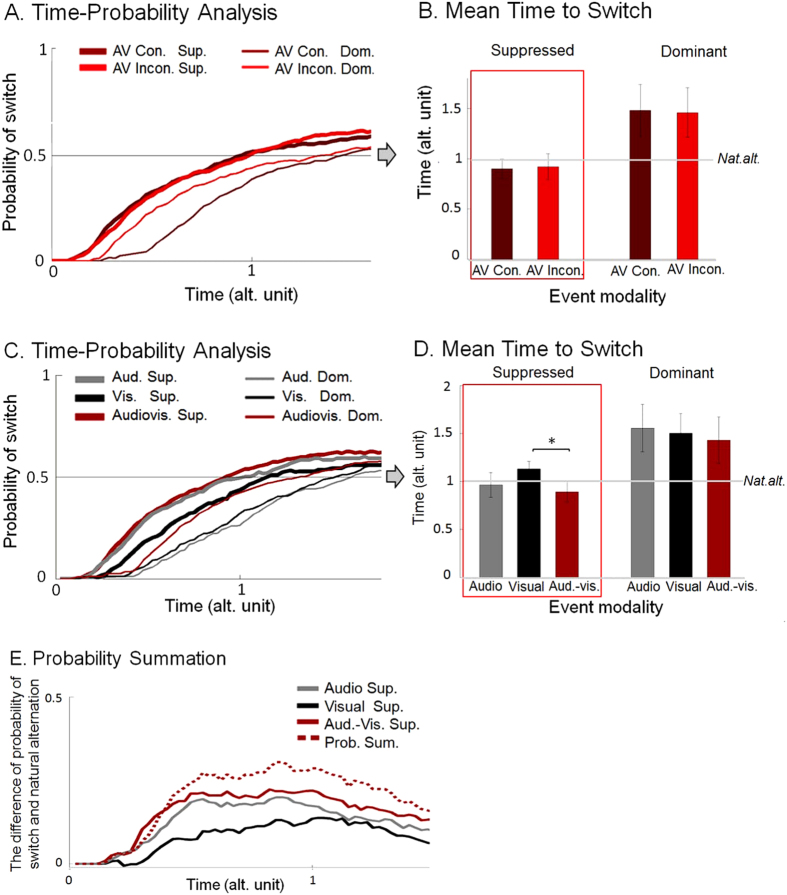
(**A**) *Time-Probability Analysis.* The probability of switch is plotted as a function of relative time, expressed in alternation units (sampling points of 0.025 alt. unit). Events were presented on Gabor patch under suppression (Audiovisual [spatially] congruent suppressed, Audiovisual incongruent suppressed) and, on Gabor patch under dominance (Audiovisual congruent dominant, Audiovisual incongruent dominant). Zero time point indicates event onset time, which never occurred during piecemeal percept. (**B**) *Mean time to switch (time at which probability crosses 50%*). The bars indicate the mean relative time of switch (in alternation unit with SEM) after in each condition separately. (**C**) *Time-Probability Analysis.* Events were presented on Gabor patch under suppression (Audio suppressed, Visual suppressed, Audiovisual suppressed, please note that in this latter case the curve calculated form the mean of congruent and incongruent conditions) and, on Gabor patch under dominance (Audio dominant, Visual dominant, Audiovisual dominant, and similarly, the given curve indicates the mean of congruent and incongruent conditions). (**D**) *Mean time to Switch (time at which probability crosses 50*%). The bars indicate the mean relative time of switch (in alternation unit with SEM) after in each condition separately. (**E**) *Probability Summation*. The curves indicate differences between different suppressed conditions (Audio suppressed, Visual suppressed, Audiovisual congruent suppressed, respectively) and the baseline alternation (probability of switch when Gabor is suppressed in natural alternation runs), as well as the curve estimated from the PSM (Prob. Sum., see Probability Summation). Additionally, please note that significant comparisons marked by ‘*’.

The main interest of the analysis of Experiment 3 data was now spatial congruency, a factor that only applies to audiovisual events. We thus compared congruent vs. incongruent audiovisual events under suppression and dominance (removing data from single modality events, i.e. visual or audio alone), with a Repeated Measures ANOVA including within participants’ factors: percept dominance (*Gabor dominant, suppressed*) and audiovisual congruency (*congruent, incongruent*). A main effect of percept dominance F(1,11) = 9.659, p = 0.01 suggested that switches happened faster when the AV event was presented on the suppressed, than on the dominant percept. However, the main effect of audiovisual congruency, F < 1, p = 0.834, and the interaction effect with percept dominance F < 1, p = 0.523 were far from significant. The main contrast of interest showed, therefore, no difference between AV congruent and incongruent conditions (Fig. 3A,B). Due to this lack of difference, we pooled the data from these two conditions in a follow-up, confirmatory analysis replicating the ANOVA of the previous two studies, which confirmed the relevant findings of Experiments 1 and 2. Even if the conditions under suppression were not faster than natural alternation, the relative pattern between suppressed conditions was replicated (i.e., faster MTS in cross-modal versus unimodal conditions, the difference between audio and audiovisual conditions remained a tendency though) (Fig. 3C–E) (see Supplemental Material, *Time-Probability Analysis: confirmatory analyses* and *Probability Summation*). We also measured spatial discrimination performance regarding sound location, after the BR sessions. Participants were able to discriminate sound location, though only when feedback was provided (indicating that, at least at a neural level, some auditory discrimination occurred but, that participants probably could not build conscious expectations about the location of sound (see Supplemental Material, *Audio localization test*).

The absence of effects derived from spatially aligned versus misaligned conditions in this experiment suggests that the lack of multisensory integration (at least, beyond statistical facilitation) in Experiment 1 could not be explained by spatial misalignment. Rather, in Experiments 1-3 spatial misalignment probably was within the tolerance limits for multisensory integration (considering that auditory receptive fields are larger than visual receptive fields, so an auditory stimulus would excite neurons over a large spatial region, including regions that are facilitated by the concurrent visual stimulus)^45^. One could perhaps argue that spatial misalignment might have produced sub-additive interactions, hence accounting for lack of evidence for integration. However, we contend that the present results are well in line with the more parsimonious account of an independent contribution of the sensory stimuli in audiovisual conditions. Following this logic, as main conclusion of the study, we claim that the absence of spatial overlap cannot account for the lack of multisensory integration effects for visual events presented below awareness in this study (i.e., in Experiments 1–3).

## Discussion

In three different experiments we have reported a cross-modal facilitation between sounds and visual events presented below the threshold of awareness, but have failed to garner conclusive evidence for cross-modal integration, above and beyond their independent contributions. Please note that in all cases, visual events exerted an effect on their own, as did the sounds, since every single stimulus has a certain stimulus-driven attentional signal^46^, but their combined presentation, despite more effective than either stimulus alone, never amounted to anything beyond what would be expected from the statistical sum of their individual contributions. This happened both when the sound consisted of an abrupt onset (Experiments 1 and 3), a gap of silence (Experiment 2), and regardless of the precise spatial co-localization of the sound and the visual event (Experiment 3).

While there is ample agreement that sounds simultaneous with visual events can enhance visual performance^42^ or eventually increase perceived luminance^47^, the explanations for sound-induced visual enhancement phenomena may be varied, and remain disputed^48^. One of the points of contention is the possible role of fast, bottom-up integration mechanisms. Such mechanism receives indirect support from the discovery of direct anatomical connections between early sensory brain areas (e.g., from audio to visual^49^), or even Superior-Colliculus-mediated improvements in simple detection tasks^50^. However, a wide range of findings suggest variations from this early multisensory integration interpretation^47^, based on other mechanisms, such as response bias^51^ or the influence of higher order mechanisms including attention, expectation and/or uncertainty reduction^52^. Here, we attempted to remove the possibility of any such higher-level or top-down mechanisms, in order to single out the capacity of the bottom-up route to produce cross-modal integration. The conclusion from the present data is that multisensory integration as indexed by the PSM violation does not occur under these stripped down conditions or, at least, it cannot be distinguished from bottom-up capture from unisensory events.

Top-down influences can, eventually, explain some previously reported cases of cross-modal effects below level of awareness. Cross-modal interactions can effect suppressed (unaware) visual stimuli via expectation, based on cross-modal correlations in some features^16^,^21^,^53^. In such cases, albeit the potential for bottom-up integration is undisputed (capitalizing on spatial location, texture, meaning, orientation, spectral variation, etc), so it is also the chance for top-down influence, and the interplay between the two systems. Indeed, these studies are invariably based on protocols where the presentation of the two sensory stimuli involved is sustained in time, leaving ample opportunity for the engagement of such top-down mechanisms. In some of these studies, one could in fact account for the multisensory advantage based on top-down attention mechanisms that do not necessarily involve integration^21^,^53^. For example, Aller *et al*.^53^ recently found cross-modal interactions when visual events were below the threshold of awareness, but the result was not conclusive of bottom-up multisensory integration because sounds were correlated with the presence of visual events in the suppressed eye, hence enabling temporal prediction, as proposed by Lippert *et al*.^52^.

Yet, in some other cases, the role of top-down influence has been carefully ruled out^17^,^20^,^22^,^32^. In the study of Zhou *et al*.^17^, suppressed visual pictures were influenced by associated odours but this cross-modal influence did not occur when association was constrained to a simple expectation (induced via instructions) without real multisensory correspondence. Similarly, BR can be biased by non-visual stimuli such as the unseen position of the participant’s hand^22^, or tactile stimuli with a selectivity for orientation that is beyond the participant’s precision when tested in an explicit discrimination task^32^. In a related example, Lunghi *et al*.^20^ reported a cross-modal effect on BR based on the combination of auditory and tactile events either of which was ineffective on its own. All these results suggest multisensory integration below the level of awareness, which derives from cross-modal feature-based congruency (in orientation, odour, proprioception,…) and does not seem to be based on top-down mechanisms.

In the current study we addressed a rather different situation, because there were no stimulus features that could, in principle, be matched across modalities, besides their mere casual, temporal coincidence. Like the BR studies with feature-based matching and sustained stimulation discussed above, we showed cross-modal facilitation effects. That is, an earlier switch to awareness of visual events presented under suppression when they were accompanied by a sound. Yet, unlike these mentioned studies, the most parsimonious interpretation of our results is that the multisensory advantage in the case of abrupt stimuli seems to arise from the statistical combination of the effects of attention grabbing saliency signals coming from each sensory channel.

Because the cross-modal facilitation found in all three experiments presented in the study cannot be conclusively tagged to cross-modal integration mechanisms, we propose the more simple explanation that saliency signals from both modalities contributed to this enhancement, but independently.

This conclusion is based on the following facts: One, stimuli in each modality separately produced the effects expected from previous findings in BR^23^,^24^,^25^. That is, flashes on the suppressed eye speeded up the switch, as did abrupt sounds (see Supplemental Material, *MTS in experimental conditions vs. natural alternation*). Second, when sounds where replaced with silent gaps, the cross-modal effects operated in the same way. Replacing onsets with offsets has been used before as a means to reduce the possibility for multisensory integration^41^ and hence, put the alternative attention hypothesis to test. Andersen & Mamassian^54^ showed that it is possible to produce as much cross-modal facilitation combining sound intensity decrements (instead of gaps) with visual increments, as with sound and visual increments. Despite this result was interpreted as multisensory integration, an attentional interpretation (proposed in their paper) cannot be ruled out either. Nonetheless the possible criticism to this logic is that the cessation of noise does not mean lack of input (absolute silence), as background room noise is rather inevitable (in our case, estimated to be 35 dB [A] by soundmeter). But if we at least consider that audio gaps are weaker stimuli than audio onsets, then by means of inverse effectiveness, a stronger multisensory response would have been expected, something that did not happen either. And, third, another symptom that the present cross-modal enhancement is based on attention capture by unspecific saliency signals, rather than cross-modal integration, is its insensitivity to spatial coincidence. The spatial rule of multisensory integration initially proposed to describe cross-modal spatial register in the pattern of neural responses in the Supperior Colliculus of animals, has been shown to apply to psychophysical detection in humans in visual-auditory protocols^7^. Albeit it is not as general as it was initially assumed^8^,^55^, it has been demonstrated that detection of masked visual stimuli improves by task-irrelevant audio stimuli within an exquisite temporal (within 100 ms) and spatial (within 16° of visual angle) selectivity. Here, we did not see this kind of selectivity, suggesting that the presence of AV facilitation but absence of multisensory integration (as indicated by the PSM benchmark) in Experiment 1, may not have its origin these spatially selective integration mechanisms.

Considering our findings in the context of current literature, we are still left with at least two interpretation challenges. On the one hand, there are several prior results suggesting cross-modal interactions with unaware visual events in patients suffering reduced, or lack of, visual awareness^3^,^7^,^8^,^9^. We think the important practical implications of these findings for rehabilitation are not at stake, but the interpretation of the principles underlying these cross-modal interactions may need to be revised.

On the other hand, another interesting interpretation paradox raised by the present findings is that cross-modal integration using unconscious visual stimuli does not seem to occur via bottom-up cross-modal mechanisms in the case of abrupt, simple signals and instead, it manifests in other paradigms using sustained stimulation with precise selectivity for complex stimulus features (spatial orientation^32^, feature-based associative pairs^17^, modality depth^20^, body posture^22^). Thus, is this discrepancy really derived from the different time-course of the stimuli presentation? Or does it rather depend on the feature-based complexity (therefore informativeness)? Or else, it is more based on the combination of the two components? Perhaps the Integrated Information Theory of Consciousness by Mudrik *et al*.^34^, adapted from the work of Tononi^33^, what has been already mentioned above, might provide a possible explanation to the existence of such selective feature-based multisensory integration effects, that do not depend on top-down modulation. That is, associations between cross-modal stimuli can operate unconsciously or with less degree of consciousness once these associations have been previously established, consciously. Temporal coincidence per se, the only possible cross-modal association in our experiments, might not provide solid enough basis to reinforce or call associated stimuli pairs. Thus, for non-associated stimuli, multisensory integration below awareness cannot be guaranteed.

## Methods

### Participants

Thirty-six naïve observers participated in the study (n = 12 in each experiment; mean age = 24.26 +/−3.126 years; 12 females overall; for age and sex distribution see Supplemental Material, *Participants*). All participants reported normal or corrected-to-normal vision, normal stereo acuity, without strong eye predominance during binocular rivalry. The participants received 10 €/hour in return for taking part in the study.

### Ethics statement

Participants gave written informed consent, and all methods were carried out in accordance with Declaration of Helsinki, under a protocol approved by the local ethics committee of the University of Pompeu Fabra (CEIC - Parc de Salut Mar).

### Apparatus and Stimuli

Visual stimuli were created using MATLAB PsychToolbox extensions, displayed on 19.8-inch CRT monitor (1024 × 768; 100 Hz refresh rate) on a grey background (13.9 cd/m^2^). Two circular rival stimuli (11.5° diameter) were presented, a horizontal Gabor patch with spatial frequency 1.2 cycles/°(17 cd/m^2^), and a radial checkerboard pattern whose luminance was adjusted individually (mean 19 cd/m^2^ across participants), both defined by a Gaussian envelope (SD = 0.13°). Both rival visual stimuli were centred on a black fixation cross (size 0.25°; luminance 3 cd/m^2^) surrounded by a grey circle (0.5°; 10 cd/m^2^). Additionally, each grating was enclosed by a black circular placeholders (0.2° in width) to facilitate stable binocular alignment. These stimuli were projected to each eye through a mirror stereoscope, giving a distance from the monitor to the eye of ~57 cm. The Gabor patch was always presented to the dominant eye. The rival stimuli were showed continuously throughout the trial, whilst test probes (abrupt visual, auditory or audiovisual events) were presented at pseudorandom moments on the Gabor patch. The visual probe consisted of a brief contrast increment (adjusted individually) on one (lower/upper) half of the grating. The sound stimulus was a 500 Hz 60 dB tone. Presentation timing of cross-modal stimuli was calibrated to 1 ms precision using a BlackBox Toolkit (Cambridge Research Systems). The observers’ head rested in a forehead-chin rest. Responses were recorded through the computer keyboard. The study was run in a dimly lit, sound attenuated test room.

### Procedure

We required the participants to covertly monitor the perceived alternation between the Gabor patch and the radial checkerboard while maintaining fixation, pressing one key to report Gabor dominant, another key to radial checkerboard dominant (hence, the Gabor suppressed) percepts, and both keys when a piecemeal percept was visible. Participants could take a break any time by releasing both of the keys. Observers participated in a 30-min test of stereoacuity in advance of four, 60-min experimental sessions, ran in five consecutive days. In Experiment 3 the last session, due to a further audio localization test took 80 mins.

### Pre-experiment adjustments and measurements

Each experimental session started with dark adaptation and calibration of the mirror stereoscope, followed by a training period where participants became acquainted with the BR paradigm. Then, the relative dominance of the radial checkerboard was set between 65% and 75% using an up-down adaptive staircase to adjust contrast of the checkerboard^56^. Next, we established the contrast level for 50% flash detection threshold (30 ms, upper or lower half on the Gabor percept under suppression), using an adaptive staircase procedure with two different delays (1000 ms or 1500 ms) of probe presentation^56^. We used 80% of this contrast value in the experiment in order to keep visual transient weak despite possible variations during the experimental session. Please note that this threshold value measured under suppression results in a strong stimulus when presented under dominance, based on the sensitivity difference under dominance and suppression (i.e., typical sensitivity loss is a range of 0.3 to 0.5 log units relative to dominance sensitivity^57^). Finally, we tracked the natural alternation dynamics for the Gabor patch and radial checkerboard rivalry for a 3-minute-session. The resulting natural alternations dynamics was used to estimate the minimum amount of time we waited after the last dominance switch, before event presentation in the experiment. To do so, we computed the half mode of duration for dominance and suppression of the Gabor patch in natural alternation, and used these values as a key-press-locked-delay to event presentation for each individual participant (see below).

### Experiment 1

**Figure 4:**
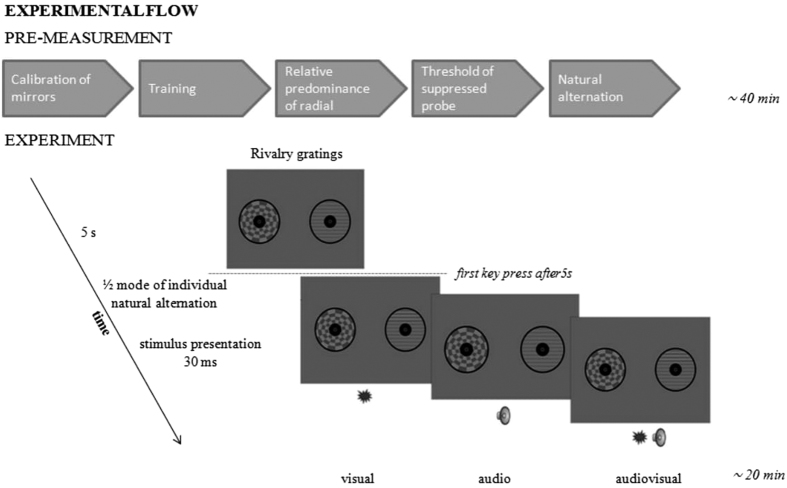
Experimental flow. Each session contained of two parts, starting with different steps of pre-measurement and followed by the experiment by itself. During the experiment each trial started with a 5-second-waiting-period. Furthermore, SOAs were fixed to an individual delay (1/2 of mode of individual natural alternation) from the first coming key-press occurred after this 5-second-period. Visual, audio or audiovisual events were presented on the dominant (D) or suppressed (S) Gabor patch. The flash is represented on the upper side of the gratings in visual and audiovisual conditions.

Observers monitored (and reported) binocular rivalry alternations between the Gabor patch and the radial checkerboard continuously, meanwhile six kinds of events were presented at pseudo-random moments. Visual only events consisted of an abrupt visual stimulus (30 ms contrast increment with 10 ms fade-in fade-out, to the upper or lower half of the Gabor, at 80% of the individual contrast threshold). This visual event could be presented when the Gabor was reported dominant (VD) or suppressed (VS). The audio-only stimulus was an abrupt tone (30 ms, 500 Hz, 60 dB(A), 10 ms fade-in and fade-out) presented from a speaker situated centrally right below the centre of the monitor. During the analysis, we separated the audio events based on whether they coincided with currently dominant (AD) or suppressed Gabor patch (AS). Audiovisual events were a conjunction of the above described visual and audio stimuli presented in temporal coincidence, creating audiovisual stimuli on the dominant (AVD) or suppressed (AVS) period of the Gabor patch. Intervals between events were adjusted individually to a key-press-locked-delay (See Procedure) added to a first coming key press occurred after a 5 s-period. If a key press happened after this 5-second-period, but before the induced individual key-press-locked-delay, the counter was restarted, therefore stimuli presentation became temporally unpredictable and uncorrelated in time (Fig. 4).

We collected data from 40 trials of each condition (type of event), although the number of audio trials was doubled, resulting in 80 collected trials. This was done to further discourage any association between visual events and sounds, and eventually allowed to divide AS and AD conditions in a post-hoc manner more effectively. As the audio-only conditions were distributed randomly in Experiment 1, the number of trials with auditory events co-occurring with Gabor suppressed (AS) and dominant (AD) periods followed the general imbalance of checkerboard predominance intended by design, and hence were similar but not exactly equal (44% vs. 56%, on average, respectively). This distribution was balanced in Experiments 2 and 3 (40 trials each, auditory dominant and auditory suppressed). Stimuli were never presented while viewers reported piecemeal percept. Participants had to report binocular dynamics without special instruction related to visual or audio stimuli. Considering the demanding nature of the rivalling monitoring and unpredictive characteristic of the events, we assume a lack of task-related top-down attentional biases toward the stimuli, other than the introspective monitoring task itself. We measured the elapsed time to a key-press after event presentation. Trials when the participant needed breaks were not included in analysis, and an event of the same condition was added to the subsequent trial run.

### Experiment 2

The experimental set-up was identical as that in Experiment 1, except for the audio. In this experiment, a constant stream of white noise (50 dB, 41000 Hz sampling rate) was interrupted by brief silent gaps which constituted the auditory stimuli. The duration of these gaps was 310 ms, starting with 10 ms fade-out and 100 ms fade-in of noise. The motivation of the time differences is to prevent to fall the onset of white noise after a gap too close to the possible visual event in audiovisual conditions.

### Experiment 3

The experimental set-up was identical as in Experiment 1, except the sounds could be presented from one of two pairs of loudspeakers aligned horizontally, and whose elevation was aligned with the location of the possible flashes. Thus, sounds could be collocated with the flashes on the monitor by using the lower or upper pair of loudspeakers at equivalent amplitude. Hence, there were four types of visual events (VD up, VD down, VS up, VS down), four types of audio events (AD up, AD down, AS up and AS down), and four types of audiovisual events (AVD [spatially] congruent, AVD incongruent, AVS congruent, AVS incongruent). This time, the sound was a noise burst (30 ms; 60 dB; 10 ms ramps). The motivation of using broadband noise instead of a pure tone was to maximize the spatial localization of the auditory event in elevation (i.e., in vertical-plane localization is more accurate for broadband noise compared to tones as wide spectral information is essential^58^). Vertical distance between speakers, 5.7°, was beyond the minimum audible angle in elevation 3.56° 59. We increased the size of visual gratings in order to maximize the distance between the spatially aligned audios, the new diameter of the visual gratings were 15.8°. Since we increased the number of experimental conditions, we had to collect more trials in order to have 40 measurements per condition. Participants were tested on 5 sessions (approx. 1.5-2 hours/session) run on different, consecutive days, the same time each day. Additionally, an audio localization test was added after the very last experimental block in order to measure whether audio stimuli in elevation were consciously discriminable (see Supplemental Material).

### Data analysis

Trials with missing data (without key press) were removed from the analysis. In fact, individual alternation times happened to be variable (in line with literature^60^). Thus, in order to smooth out these individual differences we normalized the time of each individual participant. Suppressed and dominant conditions were separately normalized to the participant’s natural alternation rate between the radial checkerboard and Gabor patch; and calculated the rest of switch latencies relative to that individual alternation unit. We then carried out two analyses, both time-locked to the onset of events.

### Time-Probability Analysis

The time-probability analysis looked at the time course of the probability of a switch in percept, time locked to event presentation, as a function of event type (Fig. 1A,B). This analysis is based on the probability of seeing dominant, suppressed and fusion percept at each sampling point (0.025 alternation unit), and is informative as to how quickly does the dominant percept changes after one of the events (presented under suppression or dominance) in the design. One useful index in this type of analysis is the Mean Time to Switch, which is the time, from event presentation, when the probability of switch surpass the probability of 50% of seeing that particular grating.

### Probability Summation

This analysis was designed to test the audiovisual data against a PSM calculated from the empirical distributions of single-modality events (auditory and visual; Fig. 1C). This test addressed the question of whether audiovisual events produce quicker switches than what one would predict simply on the basis of the sum of switching probabilities following isolated visual and auditory events. This benchmark model of summed probabilities has been used mainly on reaction times, as a test for audiovisual integration in multitude of previous studies^61^. Basically, when the summation model is violated because empirical responses to multisensory stimuli are faster than predicted, one can assume that an integration process has taken place (albeit, see^39^,^40^ for criticism).

The theoretical distribution for the PSM is calculated from the empirical cumulative distributions given by the probability that there is a switch after each type unisensory event, separately. Let the probability of switch at time T after an audio event be P(T ≤ t|S_A_), and after a visual event P(T ≤ t|S_V_). Then, one can model the theoretical audiovisual distribution based on the probability summation (R_AV_), as the probability P(T ≤ t|R_AV_) that a switch to one or the other signal has been triggered using the two unimodal distributions as follows equation (1),


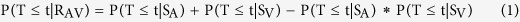


However, this estimation may be riddled by the possible problem of the double addition of variability that is unrelated to stimulus presentation. To overcome this problem, it is desirable to rule out the probability of switch when there is no stimulus. Here, this probability distribution is approximated by the empirical distribution of natural alternation (of Gabor suppressed) P(T ≤ t|S_NA_), and hence, the PSM used here is given by equation (2),


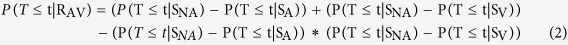


This model provides a benchmark of what would be the quickest possible switch times that one would expect under the assumption of complete independence between visual and auditory sensory processing, also called the Race Model^37^,^38^.

## Additional Information

**How to cite this article**: Pápai, M. S. and Soto-Faraco, S. Sounds can boost the awareness of visual events through attention without cross-modal integration. *Sci. Rep.* **7**, 41684; doi: 10.1038/srep41684 (2017).

**Publisher's note:** Springer Nature remains neutral with regard to jurisdictional claims in published maps and institutional affiliations.

## Supplementary Material

Supplementary Information
