# Supplementary material for: Sounds can boost the awareness of visual events through attention without cross-modal integration
Source: Sci Rep. 2017 Jan 31;7:41684. doi: 10.1038/srep41684 (PMC5282564; doi:10.1038/srep41684)
Supplement: Supplementary Information [file srep41684-s1.pdf]

SUPPLEMENTAL MATERIAL

Sounds can boost the awareness of visual events through attention without cross-modal integration

Márta Szabina Pápai and Salvador Soto-Faraco

Methods

Participants

Each of the three experiments included data from 12 observers (four, three and five females, in Experiment 1-3, respectively). The average age was 24.26+/-3.12 years (26.2 ±3.87, 24.42±3.26 and 22.16+-2.25 years in Experiment 1-3, respectively).

Audio localization test

The location and the characteristic of the audio stimuli were identical what have used during the experiment. Two blocks with 100 trials were run. Participants in a two-alternative forced choice paradigm were asked to indicate the location of the audio bursts by key press. In the first block there was no feedback provided, on the other hand, in the second block the fixation cross changed to green or red after correct or incorrect localization, respectively. The performance without feedback stayed at chance level, while with feedback it increased significantly above chance $t(11)=-5{,}016$, $p<0.01$.

Results

Experiment 1

Probability Summation tests by experiment half

In order to test whether the absence of multisensory integration can be explained by the lack of exposure to the pairing (training), we ran a test against PSM, but for data on the first versus second half of the experiment, separately. The test was just like the one performed in the whole dataset (see main text). Two-tailed, paired-t test were run on each sampling point. For the first half the empirical data was not significantly different from the threshold set up by PSM ($p>0.05$, throughout) **(Fig S1A)**. Similarly, the empirical data based on the second experiment half neither surpassed the limit of statistical facilitation indicated by PSM. A significant difference was found from 0.52 alternation unit on, although indicating that the empirical data was below the limit set by the model ($p>0.05$, throughout) **(Fig S1B)**. Therefore, these results suggest that the absence of superadditive multisensory integration cannot be explained by the lack of training (within the course of the five 1-hour-sessions, ran on consecutive days). However, please note that the "training" was never explicit. The visual flash was task-irrelevant, peripherally presented meanwhile subject conducted a highly demanding task on the alternating gratings. Due to the attentional demand on monitoring alternations, one cannot be sure whether subjects were aware of the visual stimuli under dominance, even less of the coincidence of visual and audio events. In fact, participants many times were not even aware of the task-irrelevant audio stimuli regardless its high saliency, based on subjective reports.

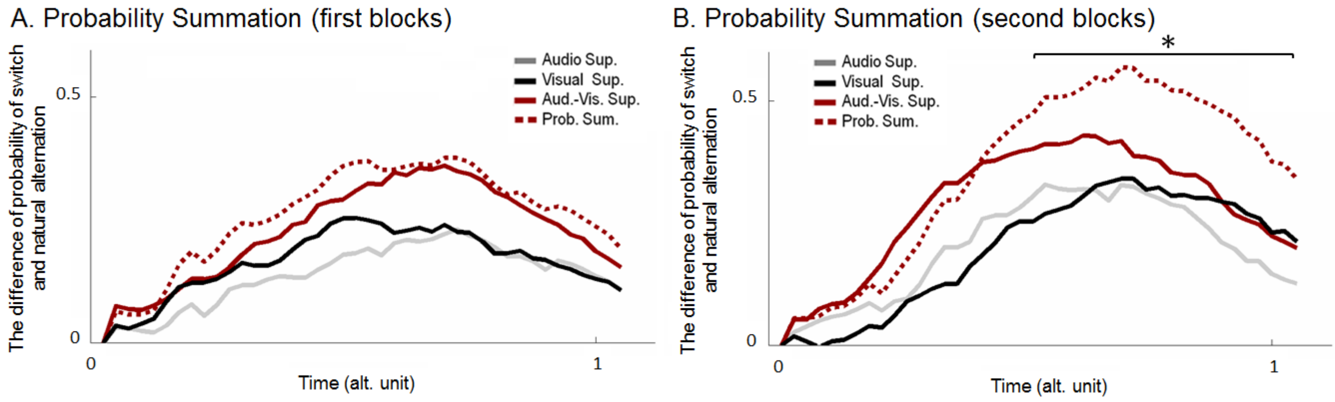

Figure S1 A. *Probability Summation (first blocks)*. The curves indicate differences between different suppressed conditions (Audio suppressed, Visual suppressed, Audiovisual suppressed, respectively) and the baseline alternation (probability of switch when Gabor is suppressed in natural alternation runs), as well as the curve estimated from the Probability Summation model (Prob. Sum.), based on the first half of the collected data **B.** *Probability Summation (second blocks)*. Curves are likewise in panel A, however now based on the data collected in the second half of the Exp1. Additionally, please note that significant comparisons marked by '*'.

MTS (Mean Time to Switch) in experimental conditions vs. natural alternation

To confirm whether our data is in line with the pattern of unimodal studies, we ran an analysis on switches in experimental condition versus in natural alternation. We conducted Repeated Measures ANOVA on percept dominance (*Gabor dominant, suppressed*) and event modality (*visual, audio, audiovisual and natural alternation*) (Greenhouse-Geisser correction was applied where needed). The ANOVA revealed a main effect of percept dominance F(1,11)=13.088, p=0.004, and importantly an interaction effect of percept dominance and event modality F(2.165,23.816)=10.106, p=0.001. This latter result let us conducted some further post-hoc analyses. As a main interest, we ran t-tests (one-tailed, paired, Bonferroni-corrected α/3=0.016) on switches in experimental conditions and natural alternation under suppression. Switches were faster in any of the experimental conditions compared to the natural switches, t(11)=-8.536, p<0.001; t(11)=-4.017, p=0.001; t(11)=-2.481, p=0.0155, in audiovisual, visual

and audio conditions, respectively. Under dominance no significant difference was found between switches in experimental conditions and natural alternation.

Experiment 3

Time-Probability Analysis: confirmatory analyses

We ran an ANOVA on percept dominance (*Gabor dominant, suppressed*) and event modality (*visual, audio, audiovisual*), where audiovisual trials contained both congruent and incongruent events (Greenhouse-Geisser correction was applied where needed). This analysis sought confirmation of the pattern of results obtained in the first two experiments. The ANOVA again revealed main effect of percept dominance $F(1,11)=8.785$, $p=0.013$ but no main effect of event modality $F(1.242,13.657)=2.286$, $p=0.151$. The critical interaction between percept dominance and event modality reached a close-to-significant level $F(1.328, 14.611)=4.105$, $p=0.052$, thus in alignment with the main result of the two previous experiments. To confirm that the data had the same pattern as in Experiments 1 and 2, we compared the MTS latencies between event modalities, the planned contrasts under suppression were measured with one-tailed paired t-tests, with Bonferroni-corrected $\alpha/2=0.025$. When events co-occurred with the suppressed Gabor, the switch happened earlier for audiovisual than for visual alone events $t(11)=-3.076$, $p=0.005$ and audio alone events, $t(11)=-1.909$, $p=0.041$, though only a tendency in this latter case. For completeness, we look at the differences between audio and visual conditions by two-tailed paired-t test, and did not occur statistically significant difference $p=0.103$.

Under dominance, MTS latency (two-tailed paired-t test with Bonferroni-corrected $\alpha/3=0.01667$) a tendency effect appeared for audiovisual versus audio events $t(11)=-2.668$, $p=0.022$, and no difference between audiovisual and visual events $p=0.492$. Between audio and visual events there was no statistically significant difference either $p=0.655$ (**Fig 3 C-D,** see main text)**.**

Furthermore, like it was the case in the two earlier experiments, switches happened faster as visual flash was presented on the suppressed grating accompanied by an audio versus visual flash was presented on the dominant grating with an audio $t(11)=-3.056$, $p=0.011$ (two-tailed paired-t, $\alpha/3=0.016$). This pattern was similar for audio events $t(11)=-3.218$, $p=0.008$, although the difference of switches after a flash on the suppressed and dominant percept remained a tendency, $t(11)=-2.258$, $p=0.045$.

Probability Summation

The empirical data were not significantly different from the threshold set up by PSM (paired-t, two-tailed, $p>0.05$, throughout) (**Fig 3E,** see main text). Furthermore, in this data set audio events had a stronger influence, as empirical probability of switch after sounds was not significantly different from the switches in audiovisual congruent condition. Anyhow, again, we cannot discard the independent contributions of stimuli as the most likely cause of cross-modal effect, suggesting a major effect of audio this time.
